# Analysis of Ancestral and Functionally Relevant CD5 Variants in Systemic Lupus Erythematosus Patients

**DOI:** 10.1371/journal.pone.0113090

**Published:** 2014-11-17

**Authors:** Maria Carmen Cenit, Mario Martínez-Florensa, Marta Consuegra, Lizette Bonet, Elena Carnero-Montoro, Noelia Armiger, Miguel Caballero-Baños, Maria Teresa Arias, Daniel Benitez, Norberto Ortego-Centeno, Enrique de Ramón, José Mario Sabio, Francisco J. García–Hernández, Carles Tolosa, Ana Suárez, Miguel A. González-Gay, Elena Bosch, Javier Martín, Francisco Lozano

**Affiliations:** 1 Instituto de Parasitología y Biomedicina López-Neyra, Consejo Superior de Investigaciones Científicas (CSIC), Granada, Spain; 2 ImmunNovative Developments, Barcelona, Spain; 3 Institut d'Investigacions Biomèdiques August Pi i Sunyer, Barcelona, Spain; 4 Institut de Biologia Evolutiva (CSIC-Universitat Pompeu Fabra), Departament de Ciències Experimentals i de la Salut, Parc de Recerca Biomèdica de Barcelona, Barcelona, Spain; 5 Department of Immunology, Hospital Clínic de Barcelona; Barcelona, Spain; 6 Department of Internal Medicine, Hospital Clínico San Cecilio, Granada, Spain; 7 Department of Internal Medicine, Hospital Carlos Haya, Málaga, Spain; 8 Department of Internal Medicine, Hospital Virgen de las Nieves, Granada, Spain; 9 Department of Internal Medicine, Hospital Virgen del Rocío, Seville, Spain; 10 Department of Internal Medicine, Hospital Parc Taulí, Sabadell, Spain; 11 Department of Functional Biology, Immunology Area, Faculty of Medicine, University of Oviedo, Oviedo, Spain; 12 Department of Rheumatology, Hospital Marques de Valdecilla, IFIMAV, Santander, Spain; 13 Departament de Biologia Cel·lular, Immunologia i Neurociencies, Facultat de Medicina, Universitat de Barcelona, Barcelona, Spain; Pavillon Kirmisson, France

## Abstract

**Objective:**

CD5 plays a crucial role in autoimmunity and is a well-established genetic risk factor of developing RA. Recently, evidence of positive selection has been provided for the *CD5* Pro224-Val471 haplotype in East Asian populations. The aim of the present work was to further analyze the functional relevance of non-synonymous *CD5* polymorphisms conforming the ancestral and the newly derived haplotypes (Pro224-Ala471 and Pro224-Val471, respectively) as well as to investigate the potential role of *CD5* on the development of SLE and/or SLE nephritis.

**Methods:**

The *CD5* SNPs rs2241002 (C/T; Pro224Leu) and rs2229177 (C/T; Ala471Val) were genotyped using TaqMan allelic discrimination assays in a total of 1,324 controls and 681 SLE patients of Spanish origin. *In vitro* analysis of CD3-mediated T cell proliferative and cytokine response profiles of healthy volunteers homozygous for the above mentioned *CD5* haplotypes were also analyzed.

**Results:**

T-cell proliferation and cytokine release were significantly increased showing a bias towards to a Th2 profile after CD3 cross-linking of peripheral mononuclear cells from healthy individuals homozygous for the ancestral Pro224-Ala471 (CC) haplotype, compared to the more recently derived Pro224-Val471 (CT). The same allelic combination was statistically associated with Lupus nephritis.

**Conclusion:**

The ancestral Ala471 *CD5* allele confers lymphocyte hyper-responsiveness to TCR/CD3 cross-linking and is associated with nephritis in SLE patients.

## Introduction

Systemic lupus erythematosus (SLE) is a systemic chronic complex autoimmune disease characterized by hyperactive T and B cells, auto-antibody production and immune complex deposition. It is considered to be the result of loss of self-tolerance triggered by certain environmental factors in genetically susceptible individuals [1]. Genetic predisposition commonly results from the combined effect of variants of a large number or genes, each allele contributing only mildly (odds ratio ∼1.5). During the past decade, the understanding of the genetic basis of SLE has been enormously expanded mainly due to genome wide association studies (GWAS). Although the loci identified in these studies as SLE genetic risk factors still account for only about 15% of the heritability of the disease [2]–[4], many have important roles in the control of innate (e.g., complement components, and interferon-inducible genes) and adaptive (e.g., major histocompatibility complex, and lymphocyte signalling effectors) immune responses [4]. Interestingly, some of these genetic factors contribute specifically to the clinical manifestations of SLE, leading to earlier onset and more severe forms of SLE [5], [6].

Extensive studies have highlighted the existence of aberrant lymphocyte function in SLE patients [7]. This may result in part from deficient control of antigen receptor-mediated signalling by regulatory molecules involved in the normal homeostasis of immune responses and/or the maintenance of self-tolerance (e.g., PD-1, CTLA-4, FcγRIIB or CD22). One of such molecules is CD5, a lymphocyte surface receptor constitutively expressed by all T cells and the B1a subset of mature B cells that has been involved in the production of low-affinity polyreactive antibodies [8] and is found expanded in patients undergoing autoimmune disorders such as rheumatoid arthritis (RA) [9], SLE [10], Sjögren syndrome (SS) [11], Grave's Basedow thyroiditis [12], and type I Diabetes Mellitus [13]. Although initially considered as a co-stimulatory molecule, the role of CD5 as a negative regulator of signalling by the antigen-specific receptor was deduced from hyper-responsiveness of thymocytes and peritoneal B1a cells from CD5-deficient mice (CD5^−/−^) to antigen-specific receptor cross-linking [14], [15]. Later work with transgenic mice expressing high- or low-affinity T-cell receptors (TCR), further indicated that CD5 expression is developmentally regulated by TCR avidity and that CD5 has a role in the fine tuning of TCR signalling [16], [17]. Notwithstanding, recent studies have revealed, a broader role of CD5 in lymphocyte biology that include regulation of activation-induced T cell death [18], [19] and recognition of pathogen-associated molecular patterns [20]. The key regulatory role played by CD5 is also supported by its physical association to the antigen-specific receptor [21], [22], as well as by its co-localization with the TCR/CD3 at the central area of the mature immune synapse, where it lowers the T cell response elicited by antigen presentation [23]. The molecular basis for the CD5-mediated negative signalling still remains unsolved, but independent studies have implicated different negative signalling effectors such as Src homology 2 domain-containing phosphatase 1 (SHP-1/PTPN6) [24], Ras GTPase activating protein (Ras-GAP) [25], cell Casitas B-lineage Lymphoma (c-CBL) [26], [27] and Casein Kinase 2 (CK2) [28]. In fact, CD5 possesses a long cytoplasmic tail devoid of intrinsic enzymatic activity but well equipped with a number of consensus motifs available to phosphorylation and/or interaction with cytoskeletal and signalling proteins [29], [30].

Consistent with its regulatory role, CD5 surface expression is found up-regulated on lymphocyte subsets with regulatory/suppressor function, namely regulatory T cells (Treg) [31] and regulatory B cells (Breg) [32], as well as on lymphocytes anergized via repeated stimulation by endogenous or exogenous antigens [33], [34].

In humans, no CD5-deficiency has been reported so far. However, a recent study has shown that an Ala→Val substitution (rs2229177, C/T) at amino acid 471 (Ala471Val), just C-terminal to an ITAM-like cytoplasmic motif, is relevant to CD5-mediated signal transduction [35]. Thus, the ancestral Ala471 variant was less efficient than the more recently derived Val471 allele in providing early biochemical signals. Interestingly, the derived allele of rs2229177*T (Val471), together with the ancestral allele (rs2241002*C, Pro224) of a second and also highly frequent nonsynonymous single nucleotide polymorphism (SNP) (rs2241002, C/T; Pro224Leu), located in the extracellular region of the CD5 molecule, was found to conform a haplotype that has been positively selected in East Asian populations [35]. This indicates the possible targeting of *CD5* polymorphisms by putative environmental factors during recent human evolution. The present work further extends the analysis of *CD5* rs2229177 and rs2241002 functional genetic variants on TCR/CD3-induced lymphocyte responses as well as on their putative involvement in autoimmunity by exploring their influence in SLE pathogenesis, either as disease-susceptibility or as disease-modifier factor.

## Materials and Methods

### SLE genetic association study

The genetic study included a total of 681 samples from SLE patients provided by different hospitals from Spain: Hospital Virgen de las Nieves and Hospital Clínico San Cecilio, (Granada), Hospital Carlos Haya (Málaga), Hospital Virgen del Rocío (Sevilla), Hospital Parc Taulí (Sabadell), Hospital Xeral-Calde (Lugo), and Hospital Central de Asturias (Oviedo), and 1,324 ethnically matched controls, all of them of European origin. All patients fulfilled the American College of Rheumatology (ACR) criteria for classification of SLE [36]. The demographic characteristics of controls and clinical features of patients included in the present study have been previously described [37]. SLE patients were classified according to the presence or absence of nephritis status. Lupus Nephritis (LN) is defined as clinical and laboratory manifestations that meet ACR criteria (persistent proteinuria and/or cellular casts including red blood cells [RBCs], hemoglobin, granular, tubular, or mixed). Written informed consent was obtained and the research followed the tenets of the Declaration of Helsinki. The local Ethics Committees of the Hospital Clínico Universitario San Cecilio (Granada, Spain), Hospital Virgen de las Nieves (Granada, Spain), Hospital Marqués de Valdecilla (Santander, Spain), Hospital Parc Tauli (Sabadell, Spain), Hospital Virgen del Rocío (Sevilla, Spain), Hospital Carlos Haya (Málaga, Spain), Hospital Xeral-Calde (Lugo, Spain) and Hospital Central de Asturias (Oviedo, Spain) approved the study.

The statistical analyses included association studies by 2×2 contingency tables and/or Fisher's exact test when necessary. *P*-values, OR and 95% CI were calculated using PLINK (V.1.07; http://pngu.mgh.harvard.edu/purcell/plink/). Hardy–Weinberg equilibrium (HWE) was tested for the studied SNPs at significance level 0.01. Allelic combinations were constructed using Haploview v4.2 by the implemented expectation–maximization algorithm. To evaluate whether the allelic combinations would better explain the possible association than the genetic variants independently, we compared the goodness of fit of both models using PLINK.

### Genotyping and SNP selection

Genomic DNA was extracted from EDTA-treated peripheral blood samples following standard procedures (QIAsymphony SP system, Qiagen). DNA samples were genotyped for the analyzed SNPs rs2241002 (C/T, Pro224Leu) and rs2229177 (C/T, Ala471Val) using predesigned TaqMan assays (Applied Biosystems, Foster City, California, USA) with IDs: C_3237272_10 and C_25472293_20. The linkage disequilibrium between the analyzed SNPs was *D′* = 0.16 and *r*2 = 0.01 in the control set.

### T-cell proliferation assays and cytokine measurements

Peripheral blood mononuclear cells (PBMC) were obtained by standard density gradient centrifugation over Ficoll (density 1.077 g/cm3; Linfosep, Biomedics) from EDTA-treated whole blood of healthy volunteers all homozygous for Pro224 (C) at SNP rs2241002 but being homozygous for either Ala471 (C) or Val471 (T) alleles at SNP rs2229177. Subjects gave informed consent before blood samples were obtained.

Proliferation assays were conducted in triplicate in round bottom 96-well plates (Costar, Corning NY, USA). PBMC (10^5^) were cultured for 72 h or 120 h at 37°C in a humidified atmosphere of 5% CO_2_ in air in a final volume of 200 µl of X-VIVO 15 (Lonza, Verviers, Belgium) in the absence or presence of different concentrations (0.1, 1.0 and 10 ng/mL) of the mouse monoclonal antibody (mAb) OKT3 (NC9983065, Ebioscience, Hatfield, United Kingdom). [^3^H] thymidine (1 µCi/well; Monavek Biochemicals, Bre, CA) was added for the last 16 h of the culture period. Then the plates were centrifuged and the supernatants collected and stored at −20°C for later determination of cytokine measurements. The cells were harvested using an automatic cell harvester and the level of [^3^H] thymidine incorporation determined in a Wallac 1205 Betaplate Liquid Scintillation Counter. Results were presented as the mean ± standard deviation (S.D.) of incorporated radioactivity, expressed in counts per minute (c.p.m.). Statistical comparisons were analyzed with two-tailed t Student test (confidence intervals of 95%) using GraphPad Prism version 4 for Windows (GraphPad Software, San Diego, CA, USA).

Cytokine levels in cell culture supernatants were assessed by using the Cytokine Human Ultrasensitive 10-Plex Panel (LHC6004, Invitrogen, Camarillo, CA, USA). Results are expressed in pg/mL as mean ± S.D. Statistical analysis was performed using a one-tailed Mann-Whitney test, with confidence intervals (CI) of 95%.

## Results

### Peripheral blood T cells from homozygotes for the Pro224-Ala471 *CD5* haplotype are hyper-responsive to TCR/CD3 cross-linking

**Figure 1:**
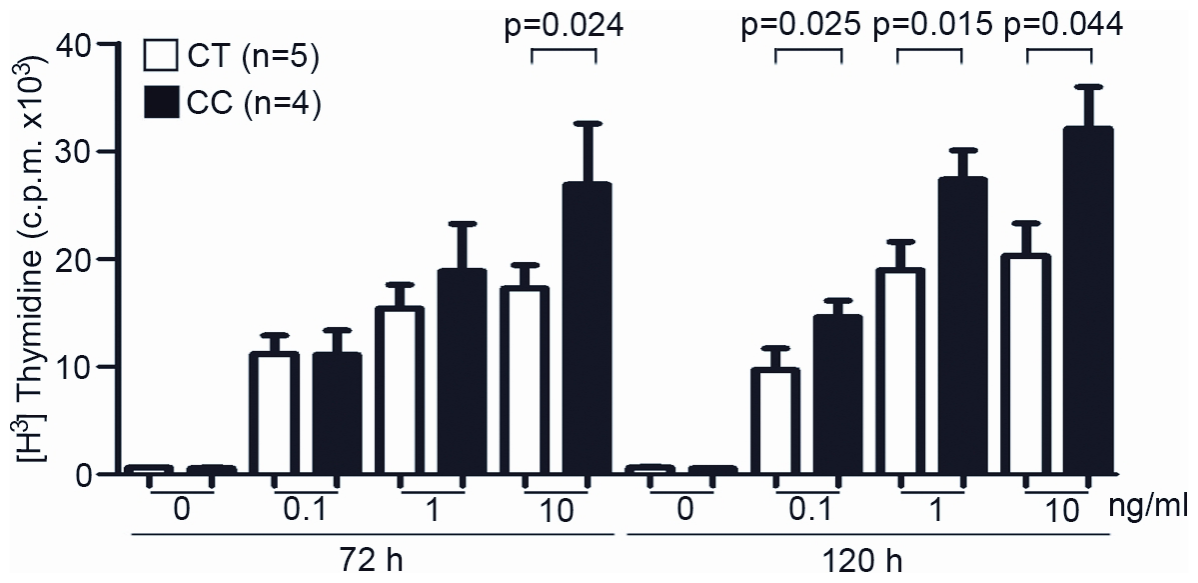
Influence of *CD5* Ala471Val polymorphism on TCR/CD3-induced peripheral T-lymphocyte proliferative responses. PBMC (10^5^ cells/well) from healthy blood donors homozygous for either the Pro224-Ala471 (CC; n = 4) or the Pro224-Val471 (CT; n = 5) haplotypes were cultured for 72 h or 120 h in the presence or absence of 0.1, 1.0 or 10 ng/mL of anti-CD3 mAb OKT3. T-cell proliferation was assessed by [^3^H] thymidine uptake and results expressed in c.p.m. as mean ± SD. Statistical analysis was performed by using two-tailed t Student test with confidence intervals of 95%.

Previous work has shown the existence of different signalling capabilities among the Ala471Val variants of the CD5 molecule when specifically cross-linked by either anti-CD5 mAb or the β-glucan rich fungal particle zymosan [35]. Accordingly, the analysis of either PBMC or cell transfectants, indicated that homozygous carriers of the ancestral Pro240-Ala471 (CC) haplotype exhibit lower intracellular signalling capability than those expressing the more recently derived Pro240-Val471 (CT) variant. Based on these results, as well as on the reported role of CD5 as negative regulator of the antigen-specific receptor signalling, it was hypothesized that expression of the Pro240-Ala471 haplotype would result in lower attenuation of activation signals generated by the T-cell receptor (TCR). To test this hypothesis, the lymphoproliferative responses to TCR/CD3 cross-linking were measured in PBMC from healthy individuals carrying the above mentioned *CD5* haplotypes. In order to reduce *CD5* genetic variability and to better ascertain the sole influence of the Ala471Val polymorphism (which was the only one showing evidence for positive selection in human populations) [35], only individuals homozygous for the ancestral Pro224-Ala471 (CC, n = 4) or the more recently derived Pro224-Val471 (CT, n = 5) haplotypes were compared. PBMC from those individuals were then exposed to different concentrations (0.1, 1.0 and 10 ng/mL) of the mouse anti-human CD3 mAb OKT3 for different periods of time (72 h or 120 h) and their proliferative capability measured by [^3^H] thymidine incorporation. As illustrated in **Figure 1**, T-cell proliferative responses at 72 h were significantly higher for Pro224-Ala471 (CC) homozygotes (p = 0.024) only when high OKT3 doses (10 ng/mL) were used. However, significant higher proliferative responses at all OKT3 concentrations tested were observed in cultures terminated at 120 h.

The influence of *CD5* Ala471Val polymorphism at the gene transcriptional level was also assessed by measuring cytokine production in culture supernatants from individuals subjected to TCR/CD3-induced T cell proliferation assays depicted in **Figure 1**. In general, increased levels were observed in Pro224-Ala471 homozygous (CC) individuals compared to Pro224-Val471 homozygous (CT) for most cytokines tested (**Table 1**). However, the differences were either at the limit of statistical significance (p = 0.057) or fully significant (p = 0.028) only for some of them (GM-CSF, IFN-γ, IL-2, IL-4, IL-5, IL-10 and TNF-α) and only at certain time points (72 h and/or 120 h).

**Table 1:** Influence of *CD5* Ala471Val polymorphism on TCR/CD3-mediated cytokine release.

	OKT3	72 h			120 h		
	(ng/mL)	Pro224Ala471 CC (n = 3)	Pro224Val471 CT (n = 4)	*p*	Pro224Ala471 CC (n = 3)	Pro224Val471 CT (n = 4)	*p*
GM-CSF	0.1	304.1±75	88.1±76.7	*	97±19.6	46.3±32.4	0.057
	1	310.1±45	108.9±67.8	*	123.6±19.1	49.4±37.2	*
	10	224±25.5	141.5±88.5	-	91.5±16.2	62.1±49.6	-
IFN-γ	0.1	426.1±103.1	94.3±97.5	*	145.7±62.8	62.2±77.5	-
	1	574.9±185	137.2±101	*	262.8±89.5	126±117.6	-
	10	510.6±196.2	412.6±184	-	228.4±97.5	124±112.8	-
IL-1β	0.1	20±4.6	15.7±2.4	-	22.4±9.7	19.9±14.6	-
	1	21.1±6	24±16	-	25.8±7.8	17.1±12.4	-
	10	18.7±3	43.2±56.2	-	24.6±9	13.2±8.5	-
IL-2	0.1	1.8±0.3	7.3±7.1	*	6.6±0.6	3.3±2.3	-
	1	2.3±0.4	17.8±27.1	-	12.1±1.8	4.1±2.7	*
	10	2.4±0.4	40.9±74.8	-	12.7±4.4	5.9±4.8	*
IL-4	0.1	11.7±1.7	10.8±8	-	12.4±1.3	6.5±6.08	-
	1	12.2±0.7	23.5±34	-	14.4±1	7.1±6.7	0.057
	10	12.3±0.9	54.3±103.6	-	13.5±1	6.8±5.7	0.057
IL-5	0.1	24.9±23.6	8±12.4	-	12.4±9.4	1.4±1.5	-
	1	36±30.7	21.1±41	-	23.1±17.9	1.5±1.1	0.057
	10	30.7±30	49.6±102.9	-	22.4±18.8	2.8±1.6	*
IL-8	0.1	13426.3±5241	7482.2±6768.8	-	19248.7±7583	10753.2±9981	-
	1	12494±6538	6703±5777	-	25819±2543.3	12455±12031	-
	10	14392.3±6585	6774.9±4481	*	20517±7240	7782.3±10661	-
IL-10	0.1	25.7±11.4	12.5±14.3	-	25.9±9.2	9.5±8.8	0.057
	1	22.3±11.1	31.8±51.1	-	33.8±9.9	10.3±9.1	*
	10	18,7±9.2	86.3±159.8	-	25.7±10	7.9±7.9	*
TNF-α	0.1	261.8±104.7	77.6±57.1	0.057	181.7±142.6	45.1±49.7	-
	1	237.1±25.2	141±93.2	-	136.3±82.9	158.7±106.8	-
	10	148.7±74.1	165.6±61.1	-	340.9±224.7	124.7±101.5	0.057

PBMCs from healthy volunteers homozygous for the rs2241002-rs2229177 *CD5* haplotypes Pro224-Ala471 (CC; n = 3) or Pro224-Val471 (CT; n = 4) were stimulated with different concentrations of OKT3 (0.1, 1.0 and 10 ng/mL) for 72 or 120 h. Cytokine levels in culture supernatants were measured in triplicate by a Luminex technique and the results expressed in pg/mL as mean ± S.D. Statistical analysis was performed using a one-tailed Mann-Whitney test, with confidence intervals of 95%. (*) full statistical significance (p<0.03). (-) non-statistically significant (p>0.05).

Taken together, the above-mentioned results indicate that peripheral T cells from carriers of the ancestral and signalling-defective *CD5* Pro224-Ala471 haplotype are hyper-responsive to TCR/CD3 cross-linking, compared to those carrying the more recently derived Pro224-Val471 allele. This is in agreement with the negative regulatory role assigned to CD5 in T cell activation [30].

### The ancestral *CD5* Pro224-Ala471 haplotype associates with SLE nephritis

Aberrant antigen receptor-mediated T-cell signalling and gene transcription responses are commonly reported in SLE patients [7]. Accordingly, an association study of the *CD5* polymorphisms was performed in a previously reported group of SLE patients [37] and a group of controls both of Spanish origin. Genotyping success rate was higher than 95% for both analyzed *CD5* SNPs (rs2241002 and rs2229177) and no statistically significant deviation from Hardy-Weinberg equilibrium (P≤0.01) was observed for any of the *CD5* SNPs studied in the control set. The genotypic and allelic frequencies of the rs2241002 and rs2229177 *CD5* SNPs in the overall SLE patients, SLE patients stratified according to the nephritis status as well as in controls are shown in **Table 2** **and** **3**. No differences between SLE patients and control individuals regarding the allelic and genotypic distribution of the analyzed *CD5* polymorphisms were observed. Nevertheless, when compared the minor allele frequencies of both analyzed *CD5* polymorphisms between SLE patients with presence of nephritis and controls statistically significant results were detected for both studied polymorphisms: rs2229177 *p* = 0.03 OR = 1.30 95% CI(1.01–1.67); rs2241002 *p* = 0.02 OR = 0.68 95% CI (0.48–0.96). On the other hand, the allelic combinations (haplotype) frequencies conformed by the studied SNPs (rs2241002-rs2229177) in overall SLE patients, SLE patients stratified accordingly to nephritis status as well as in controls were also analyzed and are shown in **Table 4**. A statistically significant increase of the *CD5* CC haplotype frequency in SLE nephritis patients compared with controls group (*p* = 7.0×10^−4^, OR = 1.52, CI 95% = 1.18–1.95) was observed. Consistently, the haplotype analysis according to the presence/absence of this clinical condition reached statistical significance (*p* = 0.016, OR = 1.42, CI 95% = 1.06–1.91). Additionally, when comparing haplotype model with the independent SNP model, a statistically significant improvement of the goodness of fit for the CC haplotype compared to rs2229177 (likelihood *p* = 0.0089) or rs2241002 (likelihood *p* = 0.015) individually was observed.

**Table 2:** Genotype and minor allele frequency of nonsynonymous *CD5* SNP rs2241002 in a Spanish cohort of SLE patients and healthy controls.

		Genotype, N (%)				Statistical Test	
SNP	Subgroup (N)	CC Pro224Pro	CT Pro224Leu	TT Leu224Leu	T Allele Frequency (%)	*P*-value	OR (CI 95%)
rs2241002	Controls (n = 1,324)	840 (63.4)	430 (32.5)	54 (4.1)	20.3		
	SLE (n = 681)	456 (67.0)	194 (28.5)	31 (4.5)	18.8	0.25	0.91 (0.77–1.07)
	SLE N+ (n = 146)	107 (73.3)	35 (24.0)	4 (2.7)	14.7	0.02	0.68 (0.48–0.96)
	SLE N− (n = 275)	182 (66.2)	81 (29.5)	12 (4.4)	19.1	0.51	0.93 (0.73–1.18)

(N+) SLE patients with nephritis;

(N−) SLE patients without nephritis.

**Table 3:** Genotype and minor allele frequency of nonsynonymous *CD5* SNP rs2229177 in a Spanish cohort of SLE patients and healthy controls.

		Genotype, N (%)				Statistical Test	
SNP	Subgroup (N)	CC Ala471Ala	CT Ala471Val	TT Val471Val	C Allele Frequency (%)	*P*-value	OR (CI 95%)
rs2229177	Controls (n = 1,324)	304 (23.0)	666 (50.3)	359 (26.7)	47.9		
	SLE (n = 681)	175 (25.7)	322 (47.3)	184 (27.0)	49.3	0.40	1.06 (0.93–1.21)
	SLE N+ (n = 146)	45 (30.8)	69 (47.3)	32 (21.9)	54.5	0.03	1.30 (1.01–1.67)
	SLE N− (n = 275)	65 (23.6)	134 (48.7)	76 (27.6)	48.0	0.98	1.00 (0.83–1.21)

(N+) SLE patients with nephritis;

(N−) SLE patients without nephritis.

**Table 4:** Allelic combinations conformed by the rs2241002-rs2229177 *CD5* SNPs in overall SLE patients, SLE patients stratified according to nephritis status (N+; N−), and controls.

Allelic combination	SLE n (%)	SLE N+ n (%)	SLE N− n (%)	*P* *	OR [95% CI]	Controls n *(%)*	*P* **	OR [95% CI]
CT Pro224Val471	512 (37.6)	101 (34.6)	214 (38.9)	0,218	0.83 [0.61–1.13]	1040 (39.3)	0.1189	0.82 (0.63–1.06)
CC Pro224Ala471	594 (43.6)	148 (50.7)	231 (42.0)	0.016	1,42 [1.06–1.91]	1070 (40.4)	7.0×10^−4^	1.52 [1.18–1.95]
TT Leu224Val471	178 (13.0)	32 (11.0)	72 (13.1)	0.371	0.82 [0.51–1.30]	334 (12.6)	0.4164	0.85 (0.57–1.27)
TC Leu224Ala471	78 (5.7)	11 (3.8)	33 (6.0)	0.166	0.61 [0.29–1.28]	204 (7.7)	0.0142	0.47 (0.24–0.90)

* P-values for the comparison SLE with nephritis (N+) versus SLE without nephritis (N−).

** P-values for the comparison SLE with nephritis (N+) versus controls.

Overall, these results support the notion that the ancestral Pro224-Ala471 haplotype (CC) is associated with Lupus nephritis, a common and severe complication of SLE. To perform a more deeply analysis, we investigated whether the individuals carrying two ancestral Pro224-Ala471 haplotype (CC) showed higher risk of developing SLE nephritis compared with the rest of the individuals. Thus, in the non CC-CC group we combined individuals Leu224/Leu224-Val471/Val471 (TT-TT) and Pro224/Leu224-Ala471/Val471 (CT-CT) since they did not show differences and we did not take into account for the analysis individuals carrying Leu224/Leu224-Ala471/Ala471 (TT-CC) and Pro224/Pro224- Val471/Val471 (CC-TT) combinations in order to avoid the influence of both polymorphisms separately on the results. Interestingly, we observed a stronger risk effect when we compared the Pro224-Ala471 homozygous individuals (CC-CC) versus Non-Pro224/Pro224-Ala471/Ala471 (Non CC-CC) frequencies between SLE with nephritis (SLE N+) and controls (P-value = 3.21×10-5 OR [95% CI] = 2.23 (1.49–3.33)) (**Table 5**). Consistently, a statistically significant difference was also observed when the same comparison was performed between SLE patients with (SLE N+) and without (SLE N−) nephritis (P-value = 7.98×10-3 OR [95% CI] = 1.88 (1.14–3.09)) while the comparison between SLE patients without nephritis (SLE N−) and controls did not reach significant results (P-value = 0.33).

**Table 5:** Frequency of combinations involving none, one and two Pro224-Ala471 haplotypes in overall SLE patients, SLE patients stratified according to nephritis (N+, N−) status and controls.

Allelic combinations	1. SLE N+	2. SLE N−	3. Controls n	*2 vs 3P value*	*1 vs 3P value*	*1 vs 2P value*
	n (%)	n (%)	*(%)*		OR [95% CI]	OR [95% CI]
A) Leu224/Leu224 + Val471/Val471 (TT-CC)	34 (23.29)	80 (29.09)	389 (29.38)	—	—	—
B) Pro224/Leu224 + Ala471/Val471 (CT-CT)	69 (47.26)	145 (52.73)	726 (54.83)	—	—	—
A+B) non CC-CC	103 (70.55)	225 (81.82)	1115 (84.21)	—	—	—
C) Pro224/Pro224 -Ala471/Ala471 (CC-CC)	43 (29.45)	50 (18.18)	209 (15.79)	0.33	3.21×10^5^ 2.2 (1.49–3.33)	7.98×10^−3^1.9 (1.14–3.09)

P-values were obtained by comparing the frequencies of Pro224-Ala471 homozygous individuals (group C) and the frequencies of Non-Pro224/Pro224 -Ala471/Ala471 individuals (groups A+B) within the different subgroups of patients and controls.

## Discussion

The present study provide the first evidence on the functional relevance of *CD5* polymorphisms in controlling the magnitude of normal human T lymphocyte responses following antigen-specific receptor triggering, as well as on their clinical relevance in autoimmunity as a putative pathogenic factor for Lupus Nephritis (LN). More precisely, it shows that peripheral T cells from healthy volunteers carrying the ancestral *CD5* Pro224-Ala471 haplotype (CC) are hyper-responsive to TCR/CD3 cross-linking compared to carriers of the more recently derived Pro224-Val471 haplotype (CT), which was boosted by natural selection in East Asian populations [35]. Moreover, the same ancestral haplotype by itself was insufficient to be associated with SLE susceptibility but with predisposition to LN in a Spanish cohort of SLE patients and controls. Therefore, this indicates that genetically determined CD5-mediated lymphocyte hyper-reactivity may act as a disease-modifier factor and result in clinical complications of SLE in conjunction with other genetic and/or environmental factors.

Carriers of the *CD5* Ala471 variant (rs2229177*C) have been reported to be less efficient than Val471 ones (rs2229177*T) in transducing early biochemical intracellular signals (namely, MAPK cascade activation) when the CD5 receptor was cross-linked via specific anti-CD5 mAbs or the β-glucan rich fungal particle zymosan [35]. In the present work, the same Ala471 variant is found associated with higher overall TCR/CD3-induced responses. This result fully fits with the negative regulatory function assigned to CD5 in TCR-signal transduction, since signalling-deficient CD5 variants (Ala471) would be expected to lower the threshold for antigen-mediated activation resulting in exacerbated T-cell proliferative responses. The fact that the different regulatory potential of *CD5* variants was evidenced without the need of co-crosslinking of CD5 with TCR/CD3 is in agreement with previous data showing that the ligation of the extracellular domains of CD5 is meaningless to this regard [38].

One of the findings worth mentioning from the present study is that two of the few cytokines giving statistically significant differences at 120 h post-stimulation were IL-10 and IL-5, two typical TH2 cytokines. SLE is considered a TH2-driven disease, although elevation of both TH1 and TH2 cytokines occurs both in human and mice, suggesting that it is a complex disease driven by different lymphocyte subsets with high heterogeneity of clinical manifestations and organ involvement [39]. Recent findings regarding LN show an essential role of TH1 and TH17 cytokines in the development of diffuse proliferative LN and of TH2 ones in that of membranous LN [39]. The histopathological characteristics of LN in our cohort are missing and it would deserve further analysis in future studies.

Of note is also the fact that the hyper-reactivity of Pro224-Ala471 haplotype (CC) carriers to TCR/CD3 cross-linking with regard to Pro224-Val471 (CT) ones is reminiscent of the phenotype reported for CD5-deficient mice [14], [15]. CD5-deficiencies have not been reported so far in human populations, and the functional *CD5* variants herein analyzed may represent adaptation responses to different environmental pressures. In fact, while the Pro224-Ala471 haplotype (CC) is well represented in human populations of African descent, it is mostly absent from East Asian populations where the more recently derived Pro224-Val471 haplotype (CT) dominates [35]. These *CD5* ethnic differences could be relevant to the pathogenesis of SLE, whose severity is known to vary among different ethnic groups. SLE has been reported to be more prevalent and severe (including LN) among African-American, Hispanic/Mestizo and Asian population groups compared to their European counterparts [40], [41]. Although the influence of lower socioeconomic status cannot be ignored [42], it is tempting to speculate whether *CD5* polymorphisms could be among the genetic factors influencing SLE ethnic patterns, at least for individuals of African ancestry where the hyper-reactive Pro224-Ala471 haplotype (CC) is prevalent.

The SLE missing heritability might be partially due to the great clinical heterogeneity of the disease. LN is a challenging problem that affects around 30–60% of SLE patients and a better understanding of its aetiology is an important step in order to identify and develop more targeted therapeutic approaches [43]. To date, no large-scale GWAS for SLE nephritis have been published. Thus, consistent with the pre-GWAS era, the literature on LN genetics has not attained the same level of maturity as it has reached in SLE [44]. Very recently, the *CD5* gene has been identified as genetic risk factor associated with RA by a high density genetic mapping approach [45]. Nowadays, it is well established that multiple disease-associated genes are shared between different autoimmune disorders [46], [47]. For instance, most of the genetic associations described for RA have been also reported to play a role in the susceptibility to SLE [47]. Moreover, preliminary results from our group indicates that CD5 polymorphism also influences the clinical expression of primary SS [48] Considering the herein presented results and the previous findings in RA and SS [45], [48], the *CD5* gene could be considered as a new shared genetic risk or disease-modifier factor in autoimmunity. Our data show that the rs2229177*C allele (Ala471) is related to inherited lower inhibitory ability of both TCR-mediated signalling and, consequently, a more increased lymphocyte proliferation. In addition, the genetic study shows that the rs2229177*C allele frequency is higher in the group of patients with SLE nephritis than in those without this trait. It is important to note that it could be suggesting that SLE patients carrying the rs2229177*C allele could present more exacerbated lymphocyte responses and, probably a more aggressive clinical outcome of the disease such as LN. On the other hand, a significant additive effect was observed between the selected polymorphisms because the most associated allelic combination with SLE nephritis was that containing the risk alleles (the CC haplotype) of the two studied SNPs. Thus, both genetic alterations together, caused by these risk alleles, could be of higher relevance than only one of these in the pathogenic mechanisms that lead to SLE nephritis. Nevertheless, further studies using dense mapping are required in order to investigate whether the associated allelic combination is the causative genetic risk factor or otherwise is in strong linkage disequilibrium with the real causal variant/s, as well as to be able to rule out the potential contribution of other genetic factors located within this region.

In conclusion, the functional data herein reported reinforce the notion of CD5 being an important modulatory molecule in T cell activation and support a role for the *CD5* gene polymorphisms in modifying the phenotypical characteristics of SLE patients, thus suggesting that CD5 might represent a potential target for future therapeutic intervention of this autoimmune condition.
